# Bi_0.9_Ho_0.1_FeO_3_/TiO_2_ Composite Thin Films: Synthesis and Study of Optical, Electrical and Magnetic Properties

**DOI:** 10.1038/s41598-019-41570-x

**Published:** 2019-03-26

**Authors:** Md. Rafiqul Islam, M. A. Zubair, M. S. Bashar, A. K. M. B. Rashid

**Affiliations:** 1 Department of Materials and Metallurgical Engineering, Bangladesh University of Engineering & Technology, Dhaka, 1000 Bangladesh; 2 Department of Glass and Ceramic Engineering, Bangladesh University of Engineering & Technology, Dhaka, 1000 Bangladesh; 3 Institute of Fuel Research & Development, Bangladesh Council of Scientific and Industrial Research, Dhaka, 1000 Bangladesh

## Abstract

A visible light active Bi_0.9_Ho_0.1_FeO_3_ nanoparticles/TiO_2_ composite thin films with different mol.% of Bi_0.9_Ho_0.1_FeO_3_ were successfully prepared via non-aqueous sol-gel method. The incorporation of 5, 10 and 20 mol.% Bi_0.9_Ho_0.1_FeO_3_ nanoparticles in the precursor solution of TiO_2_ brings modifications in the functional properties of the composite thin films. XPS analysis indicates that interdiffusion of Fe^3+^, Ho^3+^, Bi^3+^/Ti^4+^ ions through the interfaces between Bi_0.9_Ho_0.1_FeO_3_ nanoparticles and TiO_2_ matrix reduces the concentration of Ti^3+^ ions. X-ray diffraction analysis affirms that TiO_2_ and Bi_0.9_Ho_0.1_FeO_3_ retain anatase and orthorhombic phase respectively in composite films. The composite thin film containing 20 mol.% Bi_0.9_Ho_0.1_FeO_3_ nanoparticles exhibits the most prominent absorption phenomenon in visible region and has significantly reduced indirect band gap of 2.46 eV compared to that of pure TiO_2_ (3.4 eV). Hall effect measurements confirm that the resistivity of composite film increases by ∼2.33 orders of magnitude and its carrier concentration decreases by 1.8 orders of magnitude at 5 mol.% Bi_0.9_Ho_0.1_FeO_3_ nanoparticles addition compared to those of pure TiO_2_ film. Moreover, the pure film exhibits diamagnetism, whereas the composite films have both large ferromagnetic and small diamagnetic components. The findings in this research justify that the composite film can be a potential candidate for making improved photocatalyst, resistors and spintronic devices.

## Introduction

TiO_2_ as powder or thin film has been comprehensively investigated due to their excellent photo-chemical stability, low cost and non –toxicity^1^. However, pure TiO_2_ responds only to ultraviolet (UV) light which comprises only 4% of the total sunlight. The absorption occurs in the UV light due to large band gap (3.2 eV) which eventually restricts its practical application in degradation of organic pollutants such as dyes, detergents and pesticides etc^1–4^. Because of diamagnetism and low resistivity, TiO_2_ has become a concern in the field of spintronics, resistors, sensors and multistate memory devices^5,6^. These limitations have galvanized endeavors to enhance photocatalytic activity, induce magnetism and raise resistivity of TiO_2_.

One of the attempts is to substitute a portion of the titanium with different electropositive atoms. Transition metals (e.g. Fe, Mn and Cr etc) are the most commonly used constituents that induce visible light absorption^2–4^. Besides, transition metal (TM) doped TiO_2_ has exhibited improved ferromagnetism at room temperature^7,8^. Moreover, Mn and Fe dopants in TiO_2_ have increased the resistivity significantly^7,8^. Another endeavor involves substitution of oxygen with electronegative atoms (N, C and S) which aids TiO_2_ to absorb visible light efficiently^9^. However, the introduction of substitutional elements creates scattering centers which decrease the photochemical activity. It has been reported that substitutional TM serves as a center for charge carrier recombination^2^. Besides, it has been controversial as to whether Co or Fe doped TiO_2_ is a true magnetic semiconductor or if the effect is due to the clustering of a ferromagnetic second phase^10,11^. Indeed, according to these reports doped TiO_2_ has failed to exhibit magnetism to any greater extent.

Since, the physical quantities like electrical, magnetic, and optical properties come in the category of sum or product properties, many have tried to make composites of TiO_2_ to utilize this criteria^12,13^. Magnetic properties have been improved by the fabrication of TiO_2_/Fe_3_O_4_/SiO_2_ or Fe_x_O_y_-TiO_2_ composites^14,15^. Similarly the nanostructured ZnO/TiO_2_ has been found to improve the optical properties, even though, ZnO is a wide band gap semiconductor^16^. Nowadays, the visible light activity of TiO_2_ is increased by coupling it with other narrow bandgap semiconductors. The proposition is that coupled semiconductors form a hetero-junction structure which can transfer electrons from an excited small band gap semiconductor to the other attached one in the case of proper band potentials. In_2_O_3_^17^, BiFeO_3_^18^ or CdSe co-sensitized TiO_2_^19^ heterostructures have been reported for showing better visible light activity. Multiferroic BiFeO_3_ (BFO) deserves a special mention for its narrow band gap (2.2 eV) and chemical stability which have made it a suitable visible- light responsive photocatalyst^1^. The coupling of TiO_2_ with BFO has been accomplished through different synthesis processes. For instance, core-shell structured BFO/TiO_2_ by hydrolysis precipitation approach^1^, deposition of TiO_2_ on ferroelectric BFO substrate by pulsed laser deposition^20^, growth of TiO_2_ nanofibers on BFO nanoparticles by electrospinning^18^. These reports focus on enhancing visible light activity of TiO_2_. Subsequently, several studies ponder on improving electrochemical energy storage capacity and solar energy conversion efficiency of titania by anchoring BFO nanoparticles on TiO_2_^21,22^.

The magnetic and electrical properties (e.g. resistivity, carrier concentration and mobility) still remain unreported with BFO nanoparticles due to its various limitations. Literature survey indicates that BFO nanoparticles are antiferromagnetic^23^ and have higher leakage current due to formation of oxygen vacancies (OVs)^24^. Recently, doping with tri-positive rare-earth ions (R^3+^) at the Bi site has been proposed to mitigate some of the issues mentioned above^25^. Previous reports suggest that 10 mol.% Holmium (Ho^3+^) doping at Bi site increases the magnetization by 6 times and exhibits higher resistivity compared to pure BFO^25^. Since Ho^3+^ is a rare earth metal it is expected that Bi_0.9_Ho_0.1_FeO_3_ (BHFO) nanoparticles will have better absorbance in the visible region and lower band gap than BFO^23^. The aforementioned advantages make BHFO nano particles a good candidate for incorporation in TiO_2_.

TiO_2_ thin film is better candidate for spintronics, sensors and magnetic memory devices creating faster, smaller and more energy efficient devices compared to titania nanoparticles, nanowire, nanorod or nanofiber. Both undoped and doped TiO_2_ thin films have been of immense interest due to their enhanced photocatalytic activity, better electrical properties and room temperature ferromagnetism^3,7,26,27^. To the best of our knowledge the optical, magnetic and electrical properties based on incorporation of BHFO nanoparticles in TiO_2_ thin film have not been explored yet. Herein motivated by the above concerns, BHFO nanoparticle/TiO_2_ composite thin films were prepared by non-aqueous sol-gel method and their structural, magnetic, optical and electrical properties were investigated in detail. The current study revealed that composite thin films could be a more efficient visible light absorber than pure TiO_2_. Furthermore room temperature magnetism and improved electrical properties of this composite thin films have been found.

## Experimental Section

### Materials

Bismuth nitrate pentahydrate (Bi(NO_3_)_3_.5H_2_O, Merck -India), iron nitrate nonahydrate (Fe(NO_3_)_3_.9H_2_O, Merck-India), holmium nitrate pentahydrate (Ho(NO_3_)_3._5H_2_O, Sigma Adrich-USA), citric acid (C_6_H_8_O_7_, Merck -India) and ethylene glycol (C_2_H_6_O_2_, Merck -India) were used for the synthesis of BHFO nanoparticles. In this case, C_6_H_8_O_7_ acted as the chelating agent to complex the metal cations and C_2_H_6_O_2_ was added as polymerization agent^23^.

The solutions of pure TiO_2_ thin film and BHFO nanoparticles/TiO_2_ composite thin films were prepared using titanium(IV) n-butoxide (Ti (O-nBu)_4_, Aldrich-USA) as precursor and n-butyl alcohol (n-BuOH, Merck-India) as solvent. Besides, acetylacetone (C_5_H_8_O_2_, Loba chemie) was added in the solution as chelating agent to decrease the reactivity of Ti(O-nBu)_4_^28^. Glacial acetic acid (CH_3_COOH, Qualikems) was used further to begin hydrolysis via an esterification reaction.

### Synthesis of BHFO nanoparticles

BHFO nanoparticles were synthesized using a modified sol-gel method described elsewhere^23^. Shortly, for a typical 1 g BHFO powder synthesis process stoichiometric proportion of Bi(NO_3_)_3_.5H_2_O (0.003 mol), Fe(NO_3_)_3_.9H_2_O (0.0033 mol), Ho(NO_3_)_3_.5H_2_O (0.00033 mol), C_6_H_8_O_7_ (0.0067 mol) and C_2_H_6_O_2_ (10 ml) were dissolved in 400 ml deionized water. Consecutively the solution was heated under continuous stirring at 75–85 °C for 4 h to obtain gel. The gel was dried at 100 °C for 24 h in a drier to obtain precursor xerogel. The ground precursor xerogel powders were annealed at 500 °C for two hours with a heating rate of 3 °C/min to obtain BHFO nanoparticles. BHFO pellets (thickness 0.11 cm and diameter = 1.3 cm) were prepared by mixing precursor xerogel powders with PVA binder followed by pressing (5 tons pressure) and annealing at 500 °C with a heating rate of 3 °C/min to measure electrical properties.

### Preparation of pure TiO_2_ thin films and BHFO nanoparticles/TiO_2_ composite thin films

The detail synthesis process of pure TiO_2_ thin film is delineated elsewhere^28^. Briefly, first n-BuOH (0.0884 mol) and C_5_H_8_O_2_ (0.0015 mol) were mixed and, then Ti(O-nBu)_4_ (0.005 mol) was added to the solution. Subsequently this mixture was stirred for 30 min at room temperature. CH_3_COOH (0.001 mol) was slowly added into the alkoxide solution and stirred continuously for another 30 min. Concentration of the final solution was 0.5 M and its color was yellowish.

Thin films containing 5 mol.% BHFO (marked as T^95^B^5^), 10 mol.% BHFO (marked as T^90^B^10^) and 20 mol.% BHFO (marked as T^80^B^20^) were prepared using non aqueous sol-gel method. To synthesize composite films, molar amount of n-BuOH, C_5_H_8_O_2_, Ti(O-nBu)_4_ and CH_3_COOH was kept similar as that of pure thin film. Firstly 0.0442 mol of n-BuOH was taken and calculated amount of BHFO nanoparticles was mixed into it for every composite film. The whole mixture was dispersed vigorously in an ultrasonic bath for 30 min to avoid the aggregation of BHFO nanoparticles. Then C_5_H_8_O_2_, Ti(O-nBu)_4_ and rest of n-BuOH (0.0442 mol) were added into this mixture and it was stirred for 30 min on a stirring plate at room temperature. This mixture was then taken into ultrasonic bath in which CH_3_COOH was added dropwise. The final mixture was under intensive stirring for 2 h in an ultrasonic bath. The prepared mixture eventually turned into brownish color.

The brownish and yellowish solutions were spin-coated onto the 2.5 × 2 cm^2^ glass substrate at 2000 rpm for 30 s and dried at 200 °C for 10 mins. During coating brownish solution was in stirring state continuously so that BHFO nanoparticles did not settle down. Micro pipette was used to ensure that specific amount of solution was taken every time. The spin-coating and drying processes were repeated three times. The pure and composite films were annealed in static air at 500 °C for 2 h with a heating rate of 3 °C/min.

### Characterization

XPS measurements were carried out on the PHI Quantera II spectrometer and peak fitting was performed by Origin Pro. XRD (PANalytical Empyrean X-Ray Diffractometer system) was used for phase analysis of nanoparticles and thin films utilizing a Cu x-ray source (wavelength: Kα_1_ = 1.540598 Å and Kα_2_ = 1.544426 Å). Rietveld refinement was done using FULLPROF software^29^. Field emission scanning electron microscope (FESEM: JEOL, JSM, 7600F) was employed to monitor the morphologies of nanoparticles and thin films and to determine the thickness of these thin films. Energy dispersive X-ray spectroscopy (EDS) was used for mapping the elements present in the thin films. The optical properties were evaluated using UV/Vis/NIR spectrometric measurements (Perkin Elmer, Lambda 1050). To measure the magnetic property of the thin films, 5 mm × 5 mm size sample was cut from every thin film substrate. Electrical characterizations of nanoparticles were carried out by coating both sides of the pellet with silver paste and its conductivity was measured using Precision Materials Analyzer (Radiant Technologies, Inc.: P-PMF, PMF0215-377). Hall measurement was carried out using the van der Pauw configuration (HMS-3300). The four points contacts were made by indium tin alloy soldering which enabled us to obtain the carrier concentration, resistivity and mobility of thin films. The room temperature magnetic property was determined by vibrating sample magnetometer (VSM: EV-9 Microsense).

## Results and Discussion

**Figure 1:**
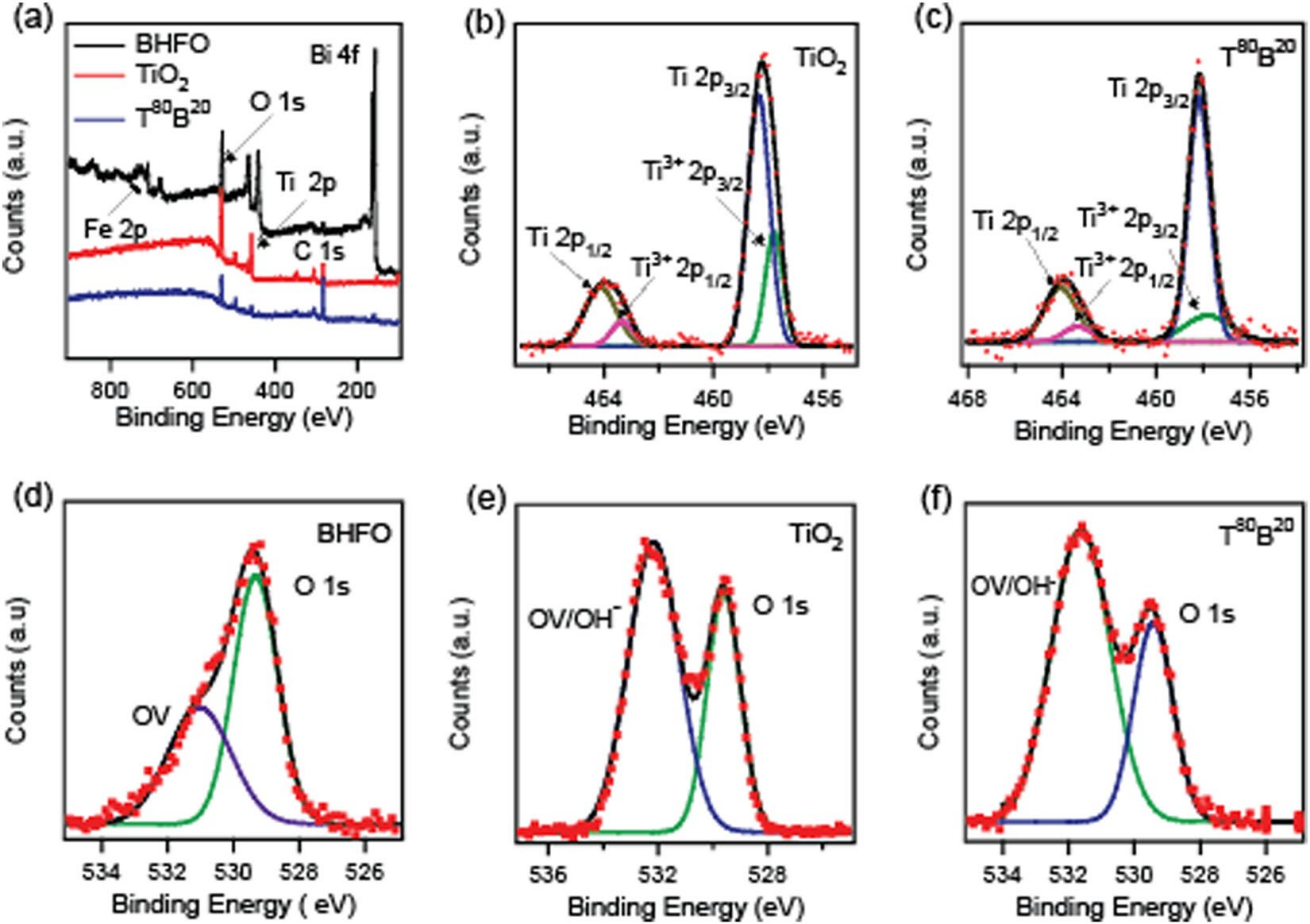
(**a**) XPS survey spectra of BHFO nanoparticles, TiO_2_ film and T^80^B^20^ composite thin film. (**b**,**c**) Ti 2p spectra of TiO_2_ and T^80^B^20^. (**d**–**f**) O 1s spectra of BHFO, TiO_2_ and T^80^B^20^.

The chemical composition and electronic structure of the as synthesized BHFO, TiO_2_ and T^80^B^20^ was determined by performing XPS analysis. Figure 1(a) depicts the XPS survey spectrum which clearly shows the presence of all the main constituent elements like Ti, Bi and O. Here, all the spectra were corrected for the C 1s peak appeared at 284.6 eV. However, Fe 2p peaks near 720 eV were not easily detectable by XPS nonetheless the EDX analysis (see Fig. S4) suggests the abundance of Fe in composite thin films. The low sensitivity of Fe to XPS analysis may lead to its absence in the survey spectra. However, the high resolution XPS scanning of BHFO nanoparticle depicts the peak corresponding to Fe having low intensity compared to other elements see Fig. S1. The high resolution elementary XPS peaks of Ti 2p and O 1s core levels observed for TiO_2_, T^80^B^20^ and BHFO after curve- fitting are presented in Fig. 1(b–f). The binding energies of the Ti 2p_3/2_ and Ti 2p_1/2_ are found to be nearly 458.4 and 464.1 eV respectively, giving a spin orbit splitting of ~5.7 eV between the two core levels, which corresponds to the 4+ oxidation state of Ti in anatase phase^30,31^. Moreover, the appearance of extra peaks at around 457.8 eV and 463.3 eV confirms the presence of Ti^3^^+ ^^31^. The peak position of Ti 2p in T^80^B^20^ has shifted negligibly. It is important to note here that the peak areas ratio for Ti^3+^ to Ti^4+^ given by (Ti^3+^ 2p_3/2_ + Ti^3+^ 2p_1/2_) _Area_/(Ti 2p_3/2_ + Ti 2p_1/2_)_Area_ in TiO_2_ and T^80^B^20^ are 0.348 and 0.231 respectively. The diminishment of the area ratio in T^80^B^20^ implies that the concentration of Ti^3+^ has decreased. The interdiffusion of A^3+^ (here A^3+^ represents Bi^3+^, Fe^3+^ and/or Ho^3+^) from BHFO to TiO_2_ across the interface could lead to a reduction in the concentration of Ti^3+^ ion. The substitution of Ti^4+^ in TiO_2_ by A^3+^ generates holes to maintain charge neutrality which oxidizes an existing Ti^3+^ to Ti ^4+^ according to the following reactions decreasing the density of electron hopping sites.

1 $${A}^{3+}\mathop{\to }\limits^{Ti{O}_{2}}{A}_{Ti}^{^{\prime} }+{h}^{\bullet }$$

2 $$T{i}^{3+}+{h}^{\bullet }=T{i}^{4+}$$

Combining Equations (1,2)

3 $${A}^{3+}+T{i}^{3+}\mathop{\to }\limits^{Ti{O}_{2}}{A}_{Ti}^{^{\prime} }+T{i}^{4+}$$

Figure 1(d) displays the XPS spectra of O 1s for BHFO, which can be deconvoluted into two peaks at around 529.4 eV and 531 eV. The lower binding energy peak is affiliated with the intrinsic O 1s core spectra and the higher energy peak is attributed to the oxygen vacant sites in BHFO. The XPS spectrum of O 1s core level for TiO_2_, as shown in Fig. 1(e), can also be de-convoluted into two symmetric Gaussian peaks centered at 529.6 and 532.2 eV^22^. The low binding energy peak at 529.6 eV of O 1s can be assigned to the −2 oxidation state of oxygen^31^. The enhancement of peak intensity at around 532 eV can be ascribed to the presence of both the oxygen vacancy related defects (OV) and the hydroxyl group (${{\rm{OH}}}^{-}$) together in T^80^B^20 ^^22,31^. The appearance of ${{\rm{OH}}}^{-}$ in TiO_2_ is normal since it is potential photocatalyst^2,4^. It is noteworthy here that the ratio of the two peak areas (OV/${{\rm{OH}}}^{-}$)_Area_/(O 1s)_Area_ in TiO_2_ and T^80^B^20^ are 1.83 and 2.20 respectively. The increment in area ratio could be due to an increment in ${{\rm{OH}}}^{-}$ group concentration in T^80^B^20^. This indicates that the composite film could be a better candidate for making improved photocatalyst than many other ceramic thin films.

**Figure 2:**
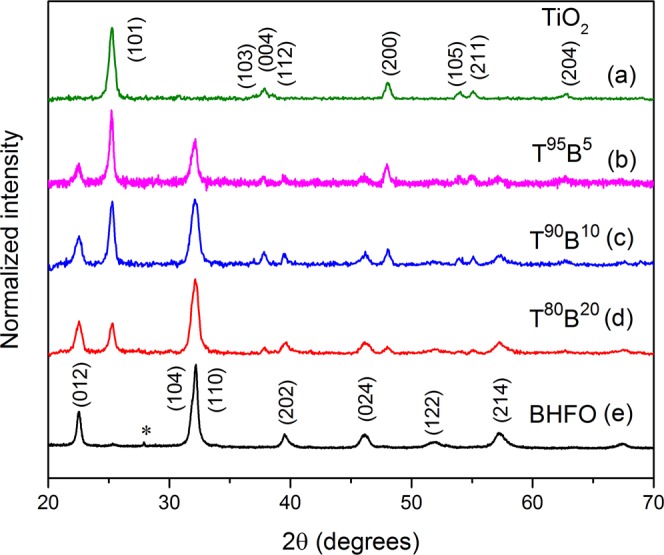
XRD patterns of the prepared pure film, composite films and nanoparticles.

Figure 2 shows room temperature XRD patterns of nanoparticles, pure thin film and composite thin films. The obtained pattern of TiO_2_ as depicted in Fig. 2(a) shows satisfactory correspondence with anatase phase. The diffraction peaks at 2θ = 25.2° (101), 37.8° (004), 48.0° (200), 53.9° (105) and 55.1° (211)) are matched well with the standard diffraction data of tetragonal crystal structure of TiO_2_^32^. Thus, it is confirmed that the annealed TiO_2_ film deposited on glass substrates has anatase phase with no trace of other polymorphs of TiO_2_ (e.g., rutile and brookite).

On the other hand, XRD pattern of BHFO as shown in Fig. 2(e) is in line with previous report of Ho^3+^ doped BFO ceramics^33^. In addition to the desired spectra one extra peak [marked by asterisk (*)] becomes visible which indicates the presence of impurity phase. To uncover the crystal structure and the impurity phase, Rietveld refinement of BHFO was carried out using the FULLPROF software^29^. The refinement corroborates that BHFO crystallizes in a orthorhombically distorted perovskite structure having Pnma space group and the extra peak in the XRD pattern belongs to Bi_2_Fe_4_O_9_ impurity phase. However, refinement indicates that Ho substituted BFO has different bond angles (Fe-O2-Fe = 151.33°, Fe-O1-Fe = 147.91°) and bond lengths (Fe-O1 = 1.49 Å, Fe-O2 = 1.82 Å) compared to that of undoped BFO^23^. The alternation of these parameters is expected to have profound influence on the magnetic and optical properties of BHFO. Weight fraction of the phases, lattice parameters and Rietveld agreement factors listed in Table S1 and fitted plot of XRD pattern as Fig. S2 are provided as Supplementary Information.

Looking at the XRD patterns for composite thin films (see Fig. 2(b–d)) it can be immediately appreciated that intensity of the peaks of BHFO increases with an increase mol.% of BHFO nanoparticles. In particular, no impurity peak can be discerned in any of the composite films. This observation solidifies that the anatase phase of TiO_2_ is retained in the films and addition of nanoparticles does not introduce any phase change. Another feature of the composite thin films is to be observed- the diffraction peaks of different planes of composite films have shifted and the lattice parameters have changed with respect to pure one (see Table 1). The positions of diffraction peaks (101) and (200) were determined using Pseudo -Voight function in high score plus software and lattice parameters were calculated using the following formulae^34^

4 $$\frac{1}{{d}_{hkl}^{2}}=[{h}^{2}+{k}^{2}+{l}^{2}(\frac{{a}^{2}}{{c}^{2}})]\frac{1}{{a}^{2}}$$

where *d*_*hkl*_ spacing has been calculated using the Bragg’s law.

**Table 1:** Position (2θ) and lattice parameters values obtained from XRD for pure film and composite films.

Compound	${\boldsymbol{P}}{\boldsymbol{o}}{\boldsymbol{s}}{\boldsymbol{i}}{\boldsymbol{t}}{\boldsymbol{i}}{\boldsymbol{o}}{\boldsymbol{n}}\,{{\boldsymbol{(}}{\bf{2}}{\boldsymbol{\theta }}{\boldsymbol{)}}}_{{\boldsymbol{(}}{\bf{101}}{\boldsymbol{)}}}$	${\boldsymbol{P}}{\boldsymbol{o}}{\boldsymbol{s}}{\boldsymbol{i}}{\boldsymbol{t}}{\boldsymbol{i}}{\boldsymbol{o}}{\boldsymbol{n}}\,{{\boldsymbol{(}}{\bf{2}}{\boldsymbol{\theta }}{\boldsymbol{)}}}_{{\boldsymbol{(}}{\bf{200}}{\boldsymbol{)}}}$	Lattice parameters	
			a = b (Å)	c (Å)
TiO_2_	25.2681	48.0128	3.7867	9.5092
T^95^B^5^	25.2907	48.0459	3.7843	9.4964
T^90^B^10^	25.2452	48.0097	3.7869	9.5136
T^80^B^20^	25.2485	47.9995	3.7877	9.5136

The peak shifting of composite films may be attributed to the diffusion of Fe^3+^, Ho^3+^ or Bi^3+^ ions into TiO_2_ across the interfaces of BHFO and TiO_2_. Table 1 shows that diffraction peaks (101) and (200) have shifted to higher angle for T^95^B^5^ and this film has the lowest lattice parameters. According to a previous study, this phenomenon manifests the introduction of tensile stresses due to the interstitial incorporation of doping ions in the crystal lattice of TiO_2_^35^. Some other previous studies also report that Fe ions can go either in octahedral interstitial sites or in the substitutional positions of TiO_2_ lattice^36,37^. Since the ionic radii of Fe^3+^ (0.785 Å) is lower than that of Bi^3+^ (1.14 Å) and Ho^3+^ (1.015 Å)^38^, Fe^3+^ ions have the highest chance of interstitial incorporation in TiO_2_. On the other hand, the diffraction peaks of T^90^B^10^ and T^80^B^20^ has shifted to the lower angle and they have larger lattice parameters compared to the pure film (see Table 1). These can be ascribed to the generation of compressive stresses as a result of substitutional incorporation of Bi^3+^ (1.14 Å), Ho^3+^ (1.015 Å) or Fe^3+^ (0.785 Å)^35,38^. The XPS analysis also supports this phenomenon. The comparatively larger ionic radii of Bi, Ho and Fe compared to Ti^4+^ (0.745 Å) ion^38^ possibly cause an expansion of the crystal lattice and concordant shift in the TiO_2_ diffraction peaks to a smaller angle. The substituted Ti^4+^ ions may have also diffused in crystal structure of BHFO.

**Figure 3:**
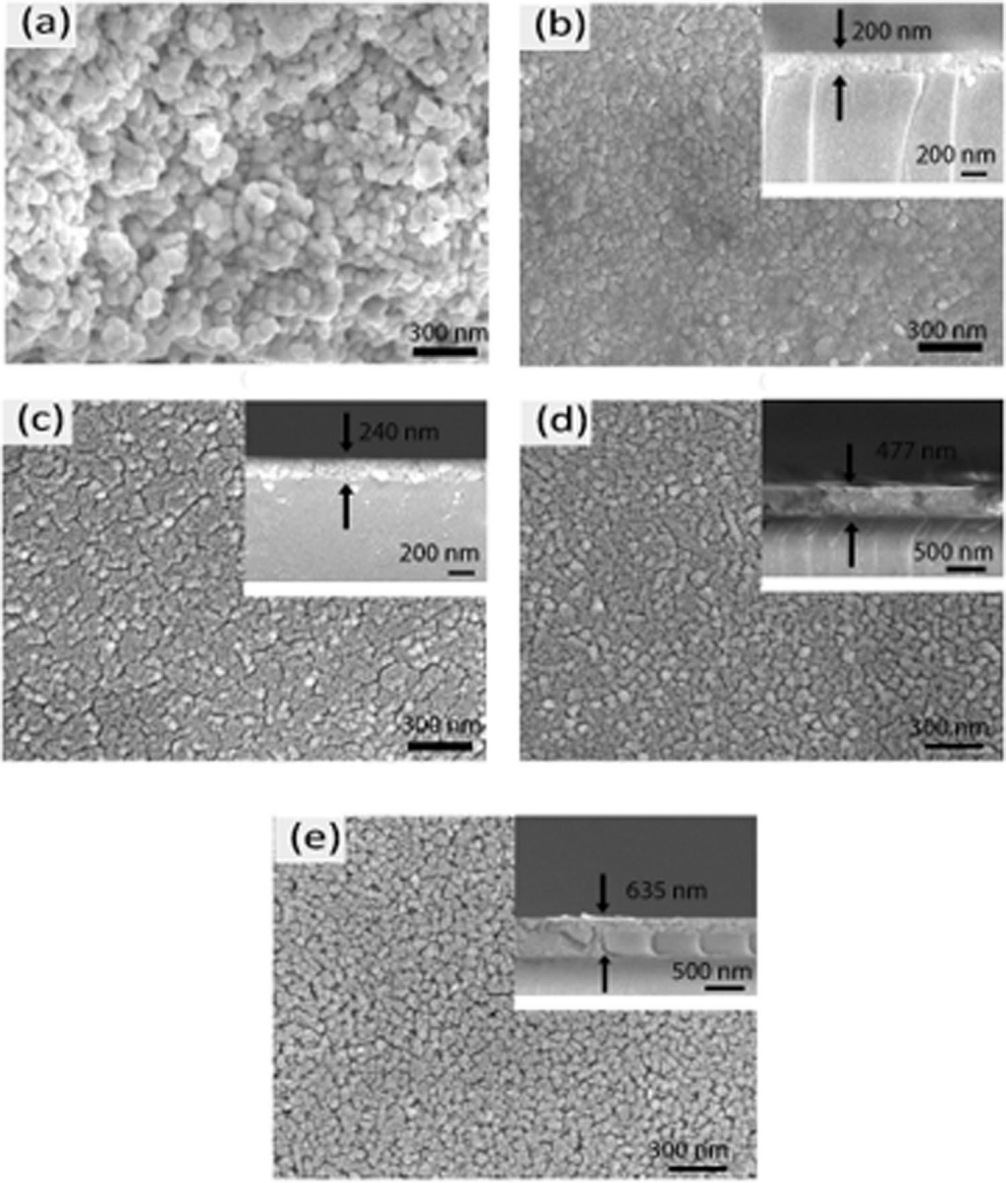
FESEM images of (**a**) BHFO nanoparticles (**b**) TiO_2_ film (**c**) T^95^B^5^ film (**d**) T^90^B^10^ film and (**e**) T^80^B^20^ film along with the films’ cross-section micrographs. The magnification for all the images is x50000.

Figure 3 exhibits the surface images of nanoparticles and films annealed at 500 °C. The observed nanoparticles are nearly spherical in shape with average diameter of 45 nm as depicted in Fig. 3(a). It can be clearly seen from Fig. 3(a) that nanoparticles are agglomerated and thus they were dispersed in an ultrasonic bath before composite thin film preparation. On the other side, the grains of pure TiO_2_ film are polygonal and crack-free (see Fig. 3(b)) signifying the fact that annealing and densification has occurred to a satisfactory degree. The average grain size of this film is larger than that of nanoparticles and is found to be ~60 nm. The average diameter of nanoparticles and grain size of thin films were determined using Image J software.

The addition of only 5 mol.% BHFO in TiO_2_ has introduced crack as well as diminished the uniformity and polygonality of the grains (see Fig. 3(c)). Further additions of 10 and 20 mol.% BHFO nanoparticles have totally changed the morphologies of thin films (see Fig. 3(d,e)). T^90^B^10^ and T^80^B^20^ films have irregular polygonal grains and spherical nanoparticles which have made the surface rough. It has become difficult to identify TiO_2_ and BHFO nanoparticles separately from Fig. 3(d,e). Indeed, T^80^B^20^ with 20 mol.% BHFO has larger number of porosities, cracks and rougher surface compared to T^90^B^10^. The FESEM surface morphology of T^80^B^20^ film is consistent with the peak intensities (2θ = 22.54°, 32.14°, 39.48°, 46.185° and 57.34°) of XRD pattern (as depicted in Fig. 2) implying high amount of BHFO nanoparticles in the TiO_2_ matrix. Moreover, the thickness of the films is increasing with increasing mol.% of BHFO nanoparticles. It can be seen from the insets of Fig. 3(b–e), the average thicknesses of TiO_2_, T^95^B^5^, T^90^B^10^ and T^80^B^10^ are 200, 244, 477, and 635 nm respectively. The cross sections of the films are also indicating the well-adherence of films with the glass substrate. Considering all these aforementioned observations it can be reasonably stated that there is no appreciable segregation of BHFO nanoparticles in the TiO_2_ matrix.

**Figure 4:**
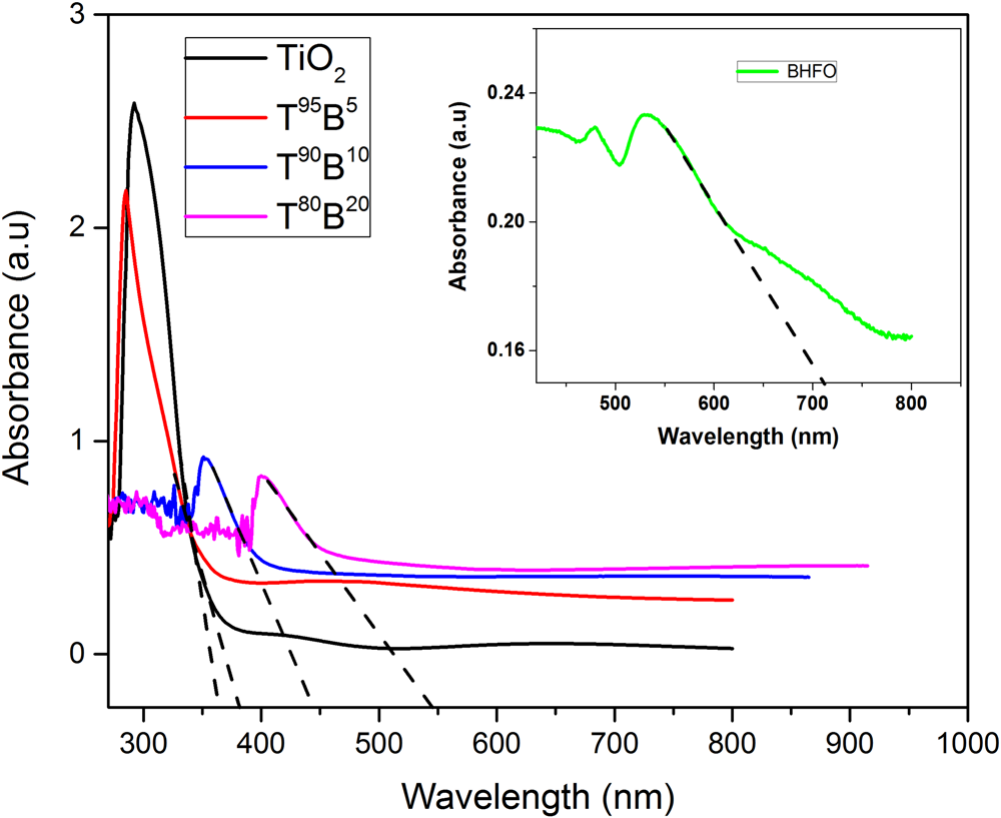
UV-vis absorbance spectra of films. The inset shows the absorbance spectra of BHFO nanoparticles.

Figure 4 depicts the absorbance spectra for thin films and nanoparticles. The absorbance of BHFO nanoparticles shown in the inset of Fig. 4 was obtained from diffused reflectance data using Kubelka-Munk conversion function^39^. The diffused reflectance data was converted to Kubelka-Munk function given by the following equation:

5 $$F(R)=\frac{{(1-R)}^{2}}{2R}$$

where R is the diffused reflectance value. On the other hand the absorbance of the films was determined using Beer- Lambert law as follows^40,41^:

6 $${\rm{A}}=log\frac{{(1-R)}^{2}}{T}$$

where A, T and R stand for the absorbance, transmittance and reflectance of the film.

Nanoparticles exhibit extensive visible light absorbance and absorb larger wavelength than the synthesized films. The steep shape of the spectrum indicates that the visible light absorption has happened as a result of electronic transition from the valence band to conduction band (O^2−^ 2p→Fe^3+^ 3d) in BHFO lattice^23^. This phenomenon will be discussed in detail in the following section. In contrast, the pure TiO_2_ film does not start absorbing substantially until the incident wavelength falls below 370 nm (see Fig. 4). This is typical behavior of TiO_2_ film^19^. It is noticeable from Fig. 4 that the absorption edge of composite thin films shows progressive shift between the pure TiO_2_ thin film and BHFO nanoparticles. Even though the absorption edge of T^95^B^5^ film does not shift much and still absorb shorter wavelength (∼380 nm) than two other composite films Fig. 4 clearly depicts that T^90^B^10^ and T^80^B^20^ films show a redshift and absorb visible light. The absorption edges of T^90^B^10^ and T^80^B^20^ films are 440 and 545 nm respectively (see Fig. 4). It has been established that the interfacial charge of TiO_2_ gets affected by diffusion of foreign ions which in turn influences the optical properties^42,43^. Indeed, the decrement of Ti^3+^ concentration in T^80^B^20^ film obtained from XPS analysis (see Table 1) suggests the possibility of A^3+^ ions diffusion in the crystal structure of TiO_2_. Therefore, the bathochromic shift of T^90^B^10^ and T^80^B^20^ films could be due to the interdiffusion of A^3+^/Ti^4+^ ions across the interfaces between BHFO and TiO_2_. In fact, due to interdiffusion of ions the electrons of these two films could be excited from the partially filled d orbitals of Fe^3+^(rather than the lower-lying O p orbitals) to the Ti 3d orbitals with lower energy visible light^1^.

**Figure 5:**
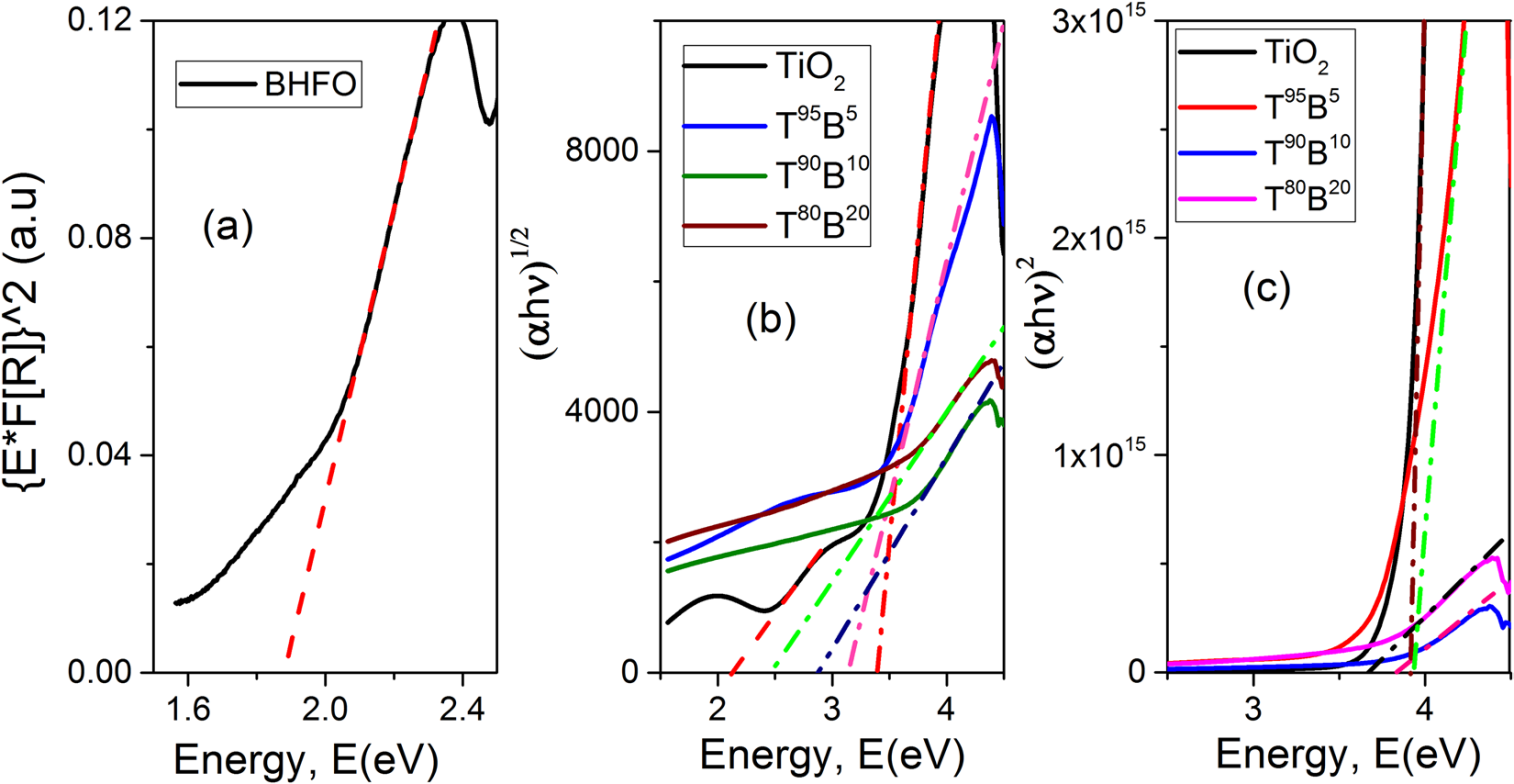
(**a**) [E*F(R)]^2^ vs energy, E plot to calculate band gap energy of nanoparticles (**b**) indirect and (**c**) direct Tauc’s plots demonstrating the band gap energies of pure and composite thin films.

In order to have better ideas on the optical properties of the films, their band gaps were determined as shown in Fig. 5. The band gap of BHFO nanoparticles obtained from [E *F(R)]^2^ vs E plot depicted in Fig. 5(a)^44^ is found to be 1.88 eV, which is notably smaller than the previously reported values for both undoped^44^ and many doped BFO^45^. The reduced band gap of BHFO could be attributed to the following reasons: (a) Ho^3+^ ions are likely to have minimal degree of hybridization for a stable electronic configuration (4f^10^ 5d^0^ 6s^0^) which in turn may lead to the formation of unique energy level in between Fe 3d and O 2p and thereby effective band gap of Ho doped BFO is diminished, (b) according to some previous investigations, changes in Fe- O bond length and Fe-O-Fe bond angle by cation doping play a pivotal role in modifying one-electron bandwidth (W) and thus band gap of BFO^23,46^. This claim has been substantiated by the empirical formula relating W with bond length and angle:

7 $$W\approx \frac{cos\omega }{{d}_{Fe-O}^{3.5}}.$$

where ω stands for ½[π- (Fe-O-Fe] and ${d}_{Fe-O}$ is the Fe-O bond length^47^. The relationship between band gap and W can be given as: ${E}_{g}={\rm{\Delta }}-W$ where Δ is the charge transfer energy^47^. Generally, BFO crystallizes in a rhombohedral phase and bond length (Fe-O) of this phase is greater than that of orthorhombic phase^23^. Since BHFO nanoparticles have orthorhombic phase and have smaller bond length, they have appreciably larger bandwidth (W) value than that of BFO which could reduce the effective band gap energy of BHFO.

The optical band gap of the films were calculated using the Tauc’s law^48^ as given below:

8 $$(\alpha h\,v)=A^{\prime} \,{(E-{E}_{g})}^{n}$$

where *A*′ is a constant, E is the photon energy, E_g_ is the band gap energy, α is the absorption coefficient and n is the power factor of the optical transition mode in a semiconductor, i.e., direct transition (n = 1/2) or indirect transition (n = 2). The band gaps of the films were obtained by extrapolating the linear portion of of (αhν)^2^ vs E plot for direct transition (see Fig. 5(c)) and (αhν)^1/2^ vs E plot for indirect transition (see Fig. 5(b)). Since the indirect band gaps of the films are more related to their absorption edge values (see Fig. 4) they have been assigned to the respective composite thin films. Even both experimental results and theoretical modeling suggest that TiO_2_ has a direct forbidden gap, which is almost degenerated with an indirect allowed transition. Therefore, the indirect allowed transition dominates in the optical absorption just above the absorption edge due to the weak strength of the direct forbidden transition^49^.

The Tauc’s plots of composite thin films corresponding to indirect transition shown in Fig. 5 have only one linear region. However, the curve for pure TiO_2_ has more than one linear regions which indicates that there is more than one optical transition occurring in this film. The first transition of pure film occurred at 3.4 eV is its effective band gap which agrees reasonably well with previous studies^35,50^. The second indirect transition occurs at 2.1 eV and it is believed that this sort of transition is attributed to the presence of OVs in deposited TiO_2_ film^19^.

**Figure 6:**
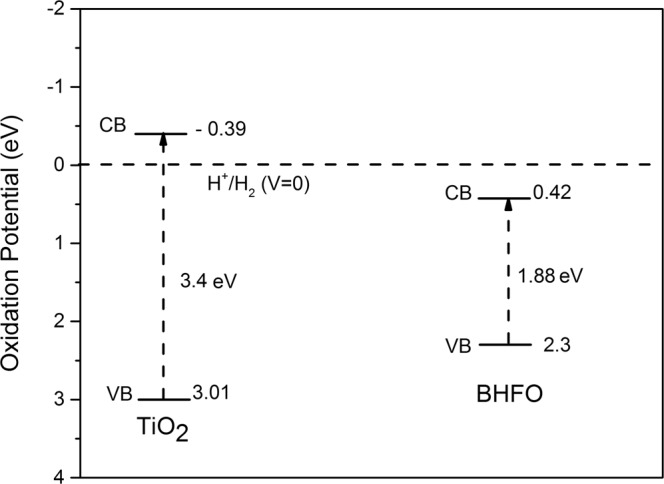
Schematic illustration for the calculated energy level diagram indicating the conduction and valence band potentials of TiO_2_ film and BHFO nanoparticles.

The indirect band gaps of T^95^B^5^, T^90^B^10^ and T^80^B^20^ films are 3.13, 2.87 and 2.46 respectively (see Fig. 5). Thus, the optical transition energies of composites thin films monotonically decrease with increasing mol.% of BHFO nanoparticles. A perceptive explanation for the reduced band gap is related to conduction band of two different semiconductors. The hypothesis is that if the conduction band edge of the sensitizing material is higher than the conduction band edge of TiO_2_, electrons can transfer from the smaller band gap material to the conduction band of TiO_2_^18,19^. According to some previous reports BFO has relatively high conduction band edge^1,51^ which makes electrons in BFO nanoparticles to be transferred easily into the lower lying CB of TiO_2_ through the interface. Thus it is required to determine the conduction band (CB) and valence band (VB) positions of both BHFO and TiO_2_ to elucidate optical transition phenomenon. The CB potential of BHFO and TiO_2_ at the point of zero charge can be calculated successfully by the following empirical equation^52,53^.

9 $${E}_{CB}={\chi }_{S}-{E}^{e}-0.5{E}_{g}$$

where *E*_*CB*_ is the CB edge potential, *E*^*e*^ is the energy of free electrons on the hydrogen scale (∼4.5 eV), *E*_*g*_ is the band gap energy of the semiconductor, *χ*_*s*_ is the electronegativity of the semiconductor, which is the geometric mean of the electronegativity of the constituent atoms^53^. The detail information on *χ*_*s*_ ihas been provided in the Supplementary Information. Plugging *E*^*e*^, *χ*_*s*_ values of TiO_2_ (5.81 eV) and BHFO (5.86 eV), and their corresponding *E*_*g*_ values into the above equations, E_CB_ of TiO_2_ and BHFO stands as −0.39 and 0.42 eV respectively. The valence band edge potential *E*_*VB*_ can be obtained by the equation: *E*_*VB*_ = *E*_*CB*_ + *E*_*g*_ and the calculated *E*_*VB*_ of TiO_2_ and BHFO are 3.01 and 2.3 eV respectively. Figure 6 depicts the calculated energy levels of TiO_2_ and BHFO.

It can be clearly seen from Fig. 6 that BHFO has relatively higher CB potential than that of TiO_2_. As discussed earlier, the redshift shown by T^90^B^10^ and T^80^B^20^ films is probably induced by Fe, Bi, Ho/Ti interdiffusion in the interfaces which could raise the CB edge potentials of BHFO and TiO_2_ and hence lower the optical transition energy at the interface between BHFO and TiO_2_. Such reduced band-gaps of T^90^B^10^ and T^80^B^20^ could be beneficial for the efficient utilization of visible light for photocatalysis. Moreover from XPS analysis a higher amount of OH^−^ has been found in T^80^B^20^ which allows the generation of large amount of highly reactive hydroxyl radicals (${{\rm{OH}}}^{\bullet }$) through oxidation of OH^−^ by photo induced holes rendering the composite film highly reactive during photocatalysis.

From the Hall effect analysis, the Hall coefficient is found to be negative for all the thin films which indicates that they are n- type semiconductors. Generally, TiO_2_ is an intrinsic n-type semiconductor^8^. The formation of the OVs and titanium interstitials are responsible for this sort of conductivity^54^. TiO_2_ is a sub-stoichiometric compound with excess titanium under standard conditions. This sub -stoichiometry is accommodated as OVs or titanium interstitials formation. The following reaction mechanisms can be considered for the formation of OVs in TiO_2_ crystal^55^:

10 $${O}_{O}^{x}\mathop{\to }\limits^{Ti{O}_{2}}\frac{1}{2}{O}_{2}(g)+{V}_{O}^{\bullet \bullet }+2{e}^{\text{'}}$$

11 $$2T{i}_{Ti}^{x}+2{e}^{\text{'}}=2T{i}_{Ti}^{\text{'}}$$

Combining Equations (4 and 5)

12 $$2T{i}_{Ti}^{x}+\,{O}_{O}^{x}=2T{i}_{Ti}^{\text{'}}+\frac{1}{2}{O}_{2}(g)+{V}_{O}^{\bullet \bullet }$$

As follows the n-type conductivity behavior found in TiO_2_ implies Equation (6) to be the dominant step for providing two excess electrons. The carrier concentration (CC), resistivity and mobility of pure TiO_2_ thin film deposited using non aqueous sol-gel method are found to be 2.0 × 10^16^ cm^−3^, 1550.8 Ω-cm and 208 cm^2^/V.s respectively. The CC of TiO_2_ found in the literatures ranges from 1 × 10^16^ cm^−3^ to 1 × 10^20^ cm^−3 ^^56,57^. Thus the measured value of CC is within accepted range. The measured mobility of the film is also consistent with the previous study which reported almost similar CC^8^. The resistivity of conductor and semiconductor normally varies from 10^−3^ to 10^8^ Ω-cm^58^. Based on this range, pure TiO_2_ film can be classified as semiconductor. However, the resistivity of this pure TiO_2_ film is lower than prepared by many methods such as chemical bath deposition^58^, spray pyrolysis^59^ and DC magnetron sputtering technique^26^.

**Figure 7:**
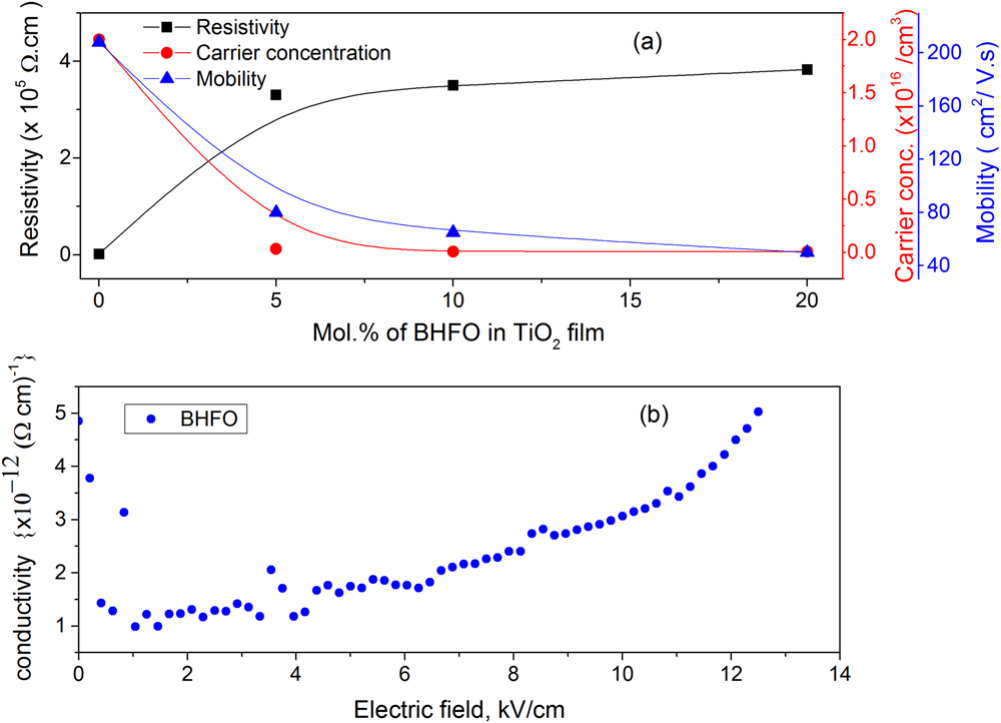
(**a**) Resistivity, carrier concentration and mobility of films with different mol.% of BHFO (**b**) Conductivity (Ω-cm) vs. Electric Field, E (kV/cm) plot of BHFO nanoparticles.

Figure 7(a) depicts that 5 mol.% BHFO addition has significantly altered the CC, mobility and resistivity of the film. The resistivity of T^95^B^5^ film is ∼2.33 orders of magnitude higher than pure film while the CC and mobility of this film are 62.5 and 2.6 times lower than those of pure one. Moreover, the charge carriers of T^95^B^5^ film are dominated by free electrons at 5 mol.% BHFO addition, even though, BFO is reported to be a p-type semiconductor where holes are the majority carriers. Here, substitution of Bi^3+^ with Ho^3+^ should retain BHFO as p-type semiconductor^60,61^. The determination of CC of BHFO was out of scope for the current study. Moreover, to the best of our knowledge there is no report on CC of BFO or on doped BFO^60,61^. Since TiO_2_ is the major phase its carrier will ultimately control the carrier concentration. The concentration of electrons may also be affected subtly by the diffused Fe ions. Since Fe ions have more than one valence states such as Fe^2+^ or Fe^3+ ^^62^, some of diffused Fe^3+^ may have changed into Fe^2+^ by accepting free electrons of TiO_2_ matrix and reduces total CC of T^95^B^5^ slightly. Thus the total CC of the composite film decreases with the addition of BHFO.

The additions of 10 and 20 mol.% BHFO have not changed the electrical properties of T^90^B^10^ and T^80^B^20^ films (see Fig. 7(a)) appreciably. The mobilities of T^90^B^10^ and T^80^B^20^ films have decreased by a factor of 1.23 and 1.6 respectively and the CCs of these two films have been reduced by a factor of ∼4 .1 when compared to those of T^95^B^5^ film. Similarly, the resistivities of T^90^B^10^ and T^80^B^20^ films are 1.05 and 1.15 times higher than that of T^95^B^5^ film. However, the charge carriers of T^90^B^10^ and T^80^B^20^ films are still found to be dominated by free electrons. The aforestated variations in CC, mobility and resistivity point out that addition of higher mol.% of BHFO has little effect on electrical properties of composite films. The trivial reduction of CC in T^90^B^10^ and T^80^B^20^ films primarily depends on diffused A^3+^ ions which substitute Ti^4+^ from the lattice sites of TiO_2_ as discussed in XPS pattern analysis section. This substitution may lead to formation of holes compensating free electrons in TiO_2_ and eventually reduces the films’ electron/Ti^3+^ concentration. However, further reduction in CC (electron hoping site concentration) is probably hindered by intergranular cracks and pore formations in the films (see Fig. 3(d,e)) as well as the direct contact interface areas between BHFO and TiO_2_ are likely to become gradually saturated at higher BHFO nanoparticles content (10 and 20 mol.%) in the composite films. These phenomena could hamper further enhancement in migration of Ho^3+^, Fe^3+^ from BHFO to TiO_2_ in T^90^B^10^ and T^80^B^20^ restricting any marked alteration in overall carrier concentration of the system.

The increased resistivity of composite thin films is directly related to low conductivity of BHFO (see Fig. 7(b)). The conductivity of BHFO varies from 1 × 10^−12^ to 5 × 10^−12^ (Ω. cm)^−1^ which is lower than that of previously reported doped BFO^23^. The CC and mobility are also related to resistivity via the following equation^63^:

13 $$\rho =\frac{1}{qn\mu }$$

where q is the electron charge, n is carrier concentration and μ is the mobility. The reduced CC and mobility of composite films could be ascribed to increased resistivity of films. Moreover, the poor morphology of the films may reduce mobility of films. Yasuno *et al*.^64^ and Trinh *et al*.^65^ observed that electrical properties of the film depend on surface roughness and porosity of film. These surface roughness and porosity increase the scattering of electrons and holes which in turn reduce their mean free path. From FESEM images it is clear that the pure film has a smoother surface than the composite films. With increasing mol.% of BHFO, the surface roughness and porosity of the films increase and charge carrier mean free path of the films decreases. Thus, the mobilities of the films decrease with reduced mean free path of electrons. The composite films with enhanced resistivity, and reduced CC and mobility will have minimum leakage current and reduced power losses in devices. Therefore, these films could be electrically reliable for resistors, sensors and memory devices.

**Figure 8:**
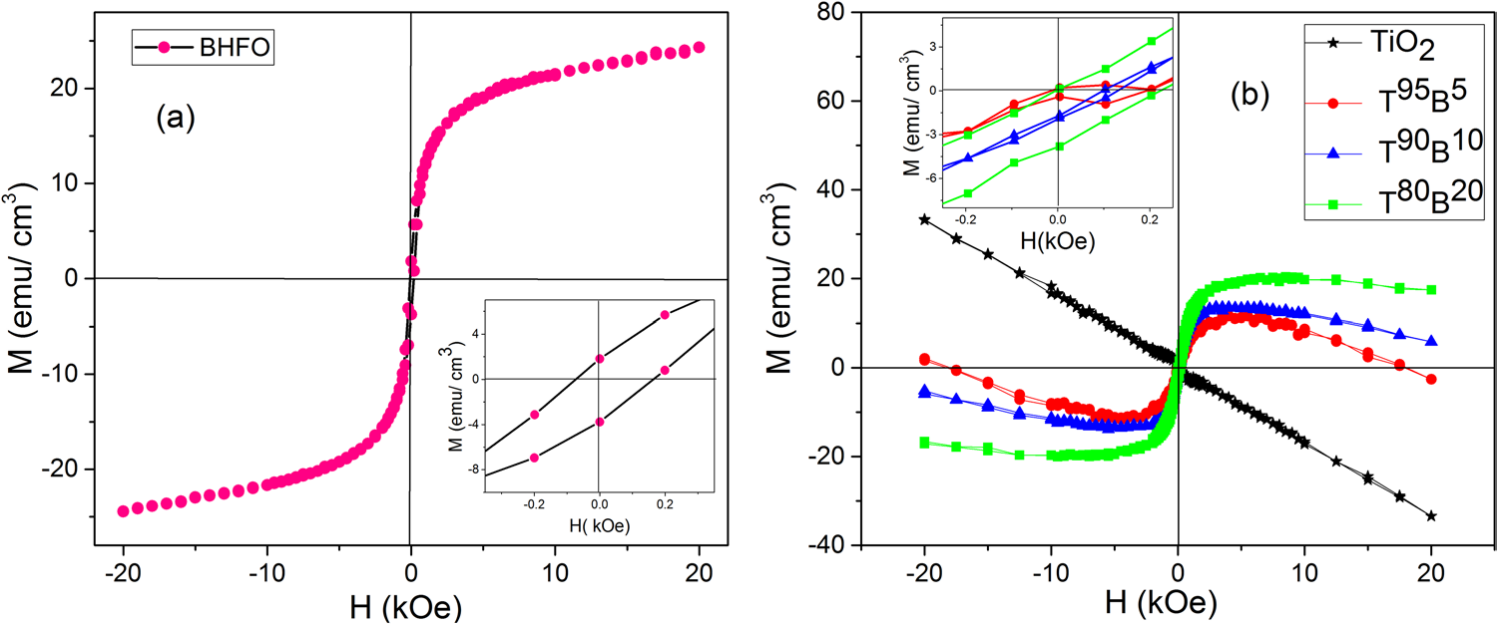
Room temperature (**a**) M-H hysteresis loop for BHFO nanoparticles with inset showing its magnified M-H loop (**b**) M-H hysteresis loops for pure and composite films with inset showing magnified M-H loops of T^95^B^5^, T^90^B^10^ and T^80^B^20^ films.

Generally BFO nanoparticles exhibit G-type antiferromagnetism due to its cycloid spiral spin structure^23^. However, an enhancement in magnetization by Ho-doping at Bi-site is evidenced in this study (see Fig. 8(a)). The saturation magnetization (Ms), coercivity (Hc) and magnetic susceptibility (*χ*_*M*_) of BHFO nanoparticles are 24.33 emu/cm^3^, 260 Oe and 0.247 respectively. The density of BHFO is 8.40 g/cm^3^ which was calculated from unit cell parameters listed in Table S1. The magnetization unit of BHFO was converted from emu/gm to emu/cm^3^ by multiplying with 8.40 g/cm^3^. The bond lengths and bond angles of BHFO listed in Table S1 are quite different from the reported bond lengths and bond angles of pure BFO^23^. The enhanced magnetization could be attributed to these changes in bond angle and bond length which modify the tilting angle of FeO_6_ octahedron and thereby suppress spiral modulated spin stricture^23^. Moreover, the substitution of non-magnetic Bi^3+^ ([Xe] 4f^14^ 5d^10^ 6 s^2^ 6p^0^) with magnetic Ho^3+^ ([Xe] 4f^10^) having high magnetic moment (10.6 μ_B_) could lead to improved magnetization.

Room temperature M-H curve of thin films were obtained after subtracting the contribution of glass substrate. TiO_2_ film deposited on this substrate is diamagnetic (see Fig. 8(b)) and the diamagnetic susceptibility (χ_*D*_′) is ∼−1.58 × 10^−03^. Donor type defects (e.g. OVs) have been proposed to be a vital element for ferromagnetism in TiO_2_^66,67^ indicating that synthesized TiO_2_ film may not have sufficient amount of OVs to induce magnetism. The other three composite thin films show no such diamagnetic behavior yet they display no complete ferromagnetic loop either. The composite thin films have ferromagnetic behavior accompanied with a diamagnetic component. The discernibly open hysteretic loop shown in the inset of Fig. 8(b) is signifying the presence of a ferromagnetic component. The actual saturation magnetization of all the composite thin films is likely to occur at low magnetic fields and all the films have diamagnetic components at higher magnetic fields. Figure 8(b) clearly depicts that saturation magnetization of the films increases with increasing mol.% of BHFO. T^80^B^20^ has higher magnetization and coercivity than two other composite films (see inset of Fig. 8(b)). Moreover, it has retained its ferromagnetism upto 11 kOe and has the lowest diamagnetic susceptibility (3.28 × 10^−4^). The diamagnetic susceptibilities of other two films are 1.01 × 10^−03^ (T^95^B^5^) and 6.045 × 10^−4^ (T^90^B^10^). Previous investigations reveal that diffusion of magnetic ions into an oxide ceramic through interfaces aids in emergence of room temperature ferromagnetism^68,69^. As conferred earlier, the interdiffusion of Fe^3+^, Ho ^3+^ and Ti^4+^ in the interfaces brings about a decrement of Ti^3+^ concentration in the crystal structure of TiO_2_. The ferromagnetic exchange between the diffused magnetic ions (Fe^3+^ or Ho^3+^) and OVs could induce magnetism^68,70^. The lacking of sufficient diffused magnetic ions to couple with OVs possibly leads to diamagnetism at higher magnetic field for the films. Since T^95^B^5^ and T^90^B^10^ have lower mol.% of BHFO compared to T^80^B^20^, they are expected to have smaller amount of diffused Fe^3+^ or Ho^3+^ ions in TiO_2_ phase than that of T^80^B^20^. Therefore, the ferromagnetic exchange of T^95^B^5^ and T^90^B^10^ is weak due to small amount of diffused magnetic ions and thus diamagnetic susceptibility of these films is higher than that of T^80^B^20^. On the other hand, T^80^B^20^ film has higher amount of magnetic ions when compared with other two composite films. Hence, a stronger ferromagnetic exchange between magnetic ions and OVs is expected in T^80^B^20^ film leading to a lower diamagnetic susceptibility compared to the other two composite films. Indeed, composites thin film with further addition of BHFO nanoparticles (>20 mol.%) will have diamagnetic component. It can be seen from Fig. S5 that the reduction in diamagnetic susceptibility from TiO_2_ film to T^95^B^5^ film is prominent but further addition of BHFO nanoparticles does not bring such kind of change. In fact Fig. S5 depicts that diamagnetic susceptibility of T^80^B^20^ film is approaching saturation phenomenon conforming our explanation provided in electrical section. The presence of pores and cracks in films (see Fig. 3(d,e)) as well as possible saturation of total amount of direct contact interface areas between BHFO and TiO_2_ not only affect the electrical properties but also the magnetic properties of T^90^B^10^ and T^80^B^20^ films. Nevertheless, this finding suggests that incorporation of BHFO nanoparticles into TiO_2_ matrix introduces magnetism in the final product which can be applied to thin films-based spintronics application.

## Conclusion

In conclusion, novel BHFO/TiO_2_ composite thin films were prepared for the first time by non-aqueous sol-gel method. The incorporation of BHFO nanoparticles improves magnetism and electrical properties of composite thin films, and also reduces their band gaps. The composite thin films exhibit a redshift in optical absorption edge and absorb visible light. Such behavior has been attributed to interdiffusion of A^3+^/Ti^4+^ ions through the interfaces between BHFO and TiO_2_. Besides, interfacial microstructural defects and the direct contact areas between BHFO and TiO_2_ in composite films control the migration of these ions through their interfaces which eventually determines the amount of CC in the films. Moreover, the ferromagnetic exchange between the diffused magnetic ions (Fe^3+^ or Ho^3+^) and OVs has been considered to influence the induction of magnetism in composite thin films.

## Supplementary information

Bi0.9Ho0.1FeO3/TiO2 Composite Thin Films: Synthesis and Study of Optical, Electrical and Magnetic Properties
