# Supplementary material for: Bi0.9Ho0.1FeO3/TiO2 Composite Thin Films: Synthesis and Study of Optical, Electrical and Magnetic Properties
Source: Sci Rep. 2019 Mar 26;9:5205. doi: 10.1038/s41598-019-41570-x (PMC6435789; doi:10.1038/s41598-019-41570-x)
Supplement: Supplementary file 1 — Bi0.9Ho0.1FeO3/TiO2 Composite Thin Films: Synthesis and Study of Optical, Electrical and Magnetic Properties [file 41598_2019_41570_MOESM1_ESM.pdf]

Supplementary Information

$Bi_{0.9}Ho_{0.1}FeO_3$/ TiO_2 Composite Thin Films: Synthesis and Study of Optical, Electrical and Magnetic Properties

Md. Rafiqul Islam[1, **], M. A. Zubair[2] , M. S. Bashar[3] and A. K. M. B. Rashid[1, *]

[1] Department of Materials and Metallurgical Engineering, Bangladesh University of Engineering & Technology, Dhaka- 1000, Bangladesh.

[2] Department of Glass and Ceramic Engineering, Bangladesh University of Engineering & Technology, Dhaka -1000, Bangladesh.

[3] Institute of Fuel Research & Development, Bangladesh Council of Scientific and Industrial Research, Dhaka- 1000, Bangladesh.

*Corresponding author: rashid.akmb@gmail.com

**Corresponding author: mrisagor@mme.buet.ac.bd

XPS analysis

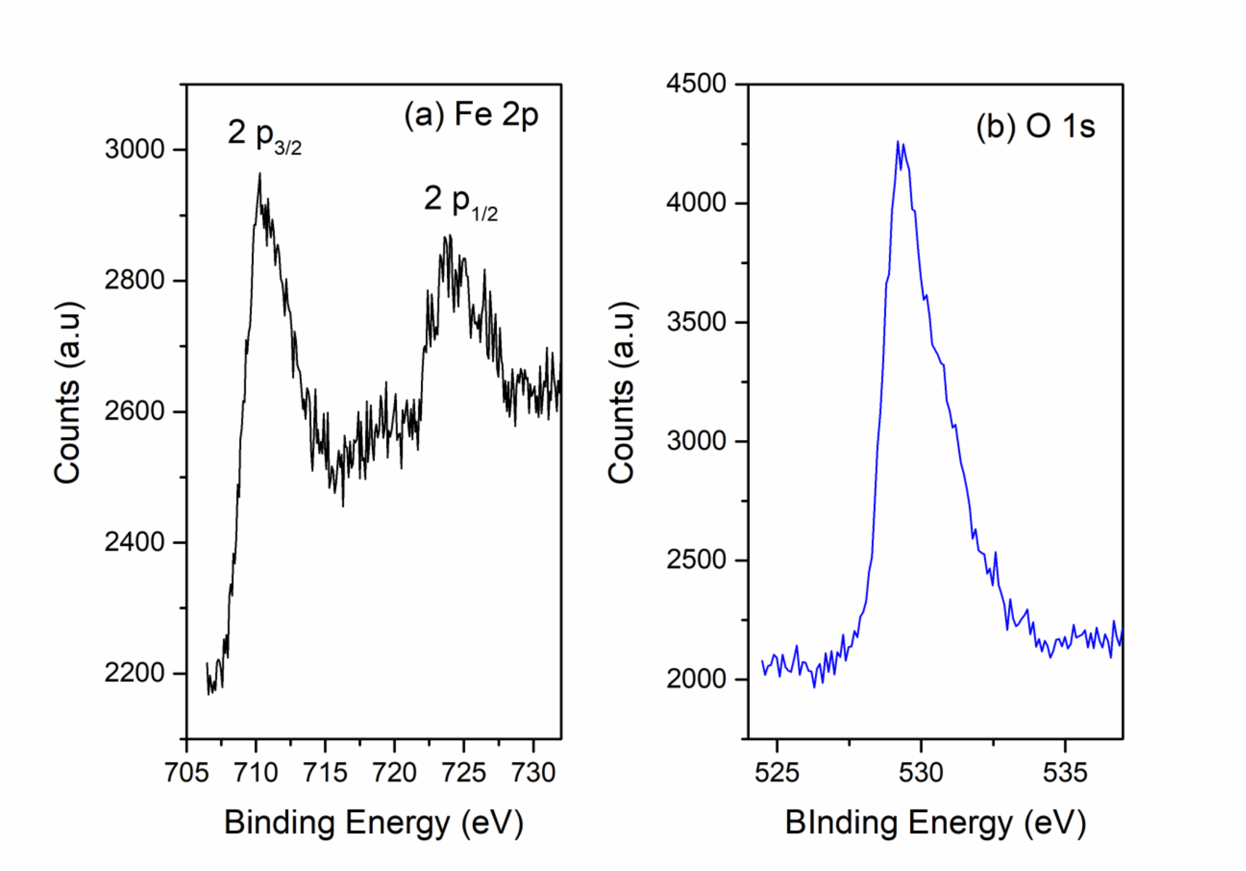

Figure S1. XPS spectra of (a) Fe 2p and (b) O 1s core levels of the BHFO nanoparticles

Figure S1 depicts the core level spectra of Fe 2p and O 1s of BHFO nanoparticles. The XPS spectra clearly indicates that peak intensity of Fe is relatively lower than that of O' s peak intensity. That is why Fe 2p peaks near 720 eV were not easily detectable by XPS in $T^{80}B^{20}$ for its low intensity.

XRD analysis

Generally, pure $BiFeO_3$ (BFO) crystallizes in a rhombohedrally distorted perovskite structure, which belongs to the space group R3c. The perovskite structure of BFO transforms from rhombohedral (R3c) to tetragonal (P4 mm) or orthorhombic (Pnma) or cubic (Pm-3m) phase

depending on the nature of the A site and B site doping.[1 2,3] As a result, diversified structural reports are available for $Bi_{0.9}Ho_{0.1}FeO_3$ (BHFO) in which the neighboring peaks such as (012), (110)/(104), (006)/(202), (116)/(112) and (018/214) are merged together.[4,5] In current study, it can be appreciated by observing the XRD patterns in Fig. S2 that substantial structural modification has occurred in the Ho substituted BFO nanoparticles. Magnified portions of the XRD patterns (see Fig. S3) for the ranges of 2θ=21.5°-23° and 2θ=30°-34° there is partial merging of (110)/(104) peaks, which immediately indicate a possible structural transition in the synthesized BHFO nanoparticles. This possible merging due to ion doping has been reported in previous studies as an indication of rhombohedral to orthorhombic or tetragonal phase transition.[6] XRD patterns collected for all the sample have been fitted by the Rietveld method using FULLPROF.[7] Rietveld refinement was carried out for rhombohedral, tetragonal, triclinic, monoclinic and orthorhombic structure. The Goodness of Fit (GoF) of our sample with orthorhombic structure (space group -Pnma) and with a second phase $Bi_2Fe_4O_9$ (space group–Pbam) has been found to be ~1.44 indicating the reliability of the refinement. Indeed, rare earth metal doped BFO crystallizes with orthorhombic structure.[4,5]

.

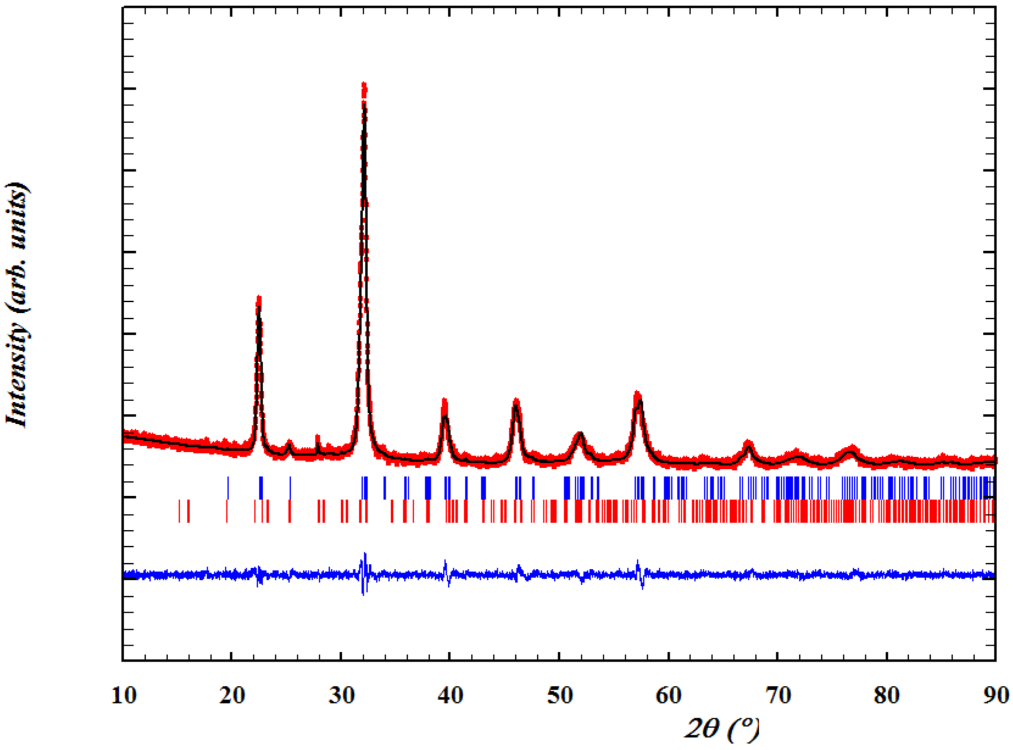

Figure S2. Plot showing the observed (red dot), calculated (black line), and difference (blue line) patterns obtained from Rietveld refinement. First and second row of ticks indicates Bragg positions for the Pnma phase and Pbam phase respectively.

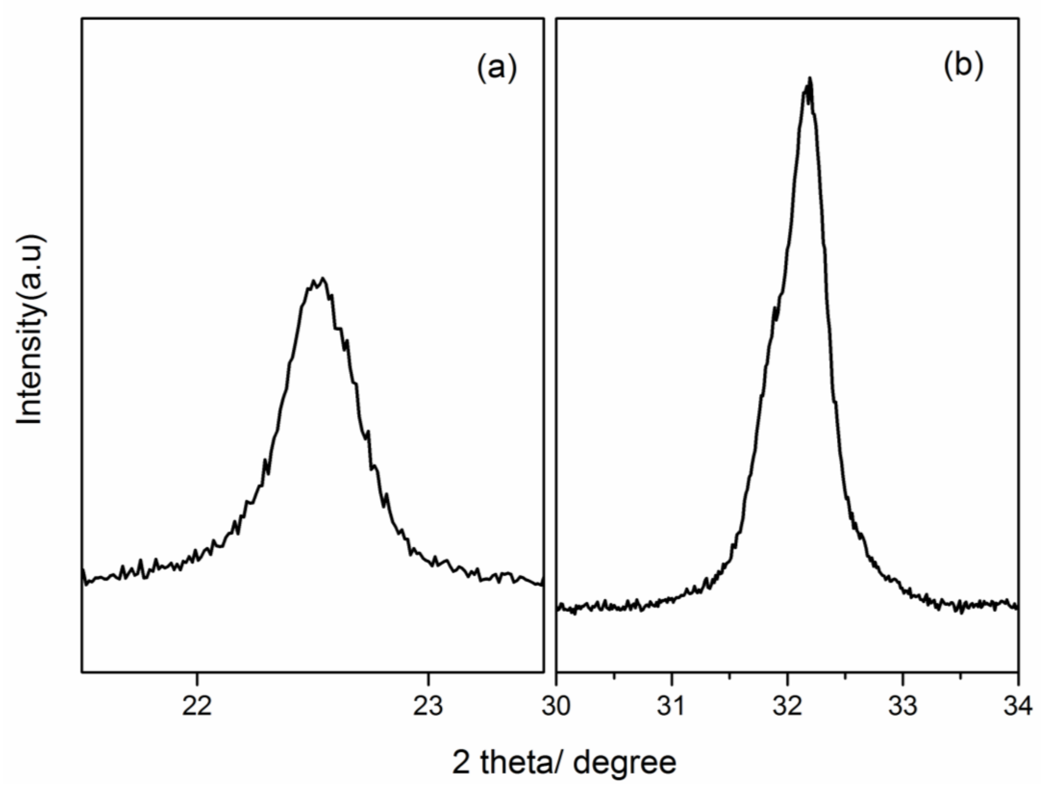

Figure S3. Magnified XRD patterns of BHFO nanoparticles in the range of (a) 2theta/degree = 21.5-23.5° and (b) 2theta/degree = 30- 34°.

Table S1: Summary of data obtained from Rietveld refinement and agreement factors of BHFO nanoparticles.

Sample	Phase(s) present	Unit cell parameters	Bond length (Å)	Bond angle	Rietveld factors
BHFO	Pnma<br>91.42 %	a=5.544209 Å<br>b= 5.614142 Å<br>c= 7.835147 Å<br>V = 243.9 $Å^3$	B-O1=2.18<br>B-O2=2.12<br>Fe-O1=1.49<br>Fe-O2=1.82	Fe-O1-Fe=147.91<br>Fe-O2-Fe=151.33	R_p= 19.6<br>R_{wp} = 15.7<br>R_{exp} = 13.1<br>χ^2 =1.441
	Pbam<br>$Bi_2Fe_4O_9$<br>8.58 %	a= 7.6526 Å<br>b =9.1057 Å<br>c=5.5454 Å<br>V = 386.415 $Å^3$			

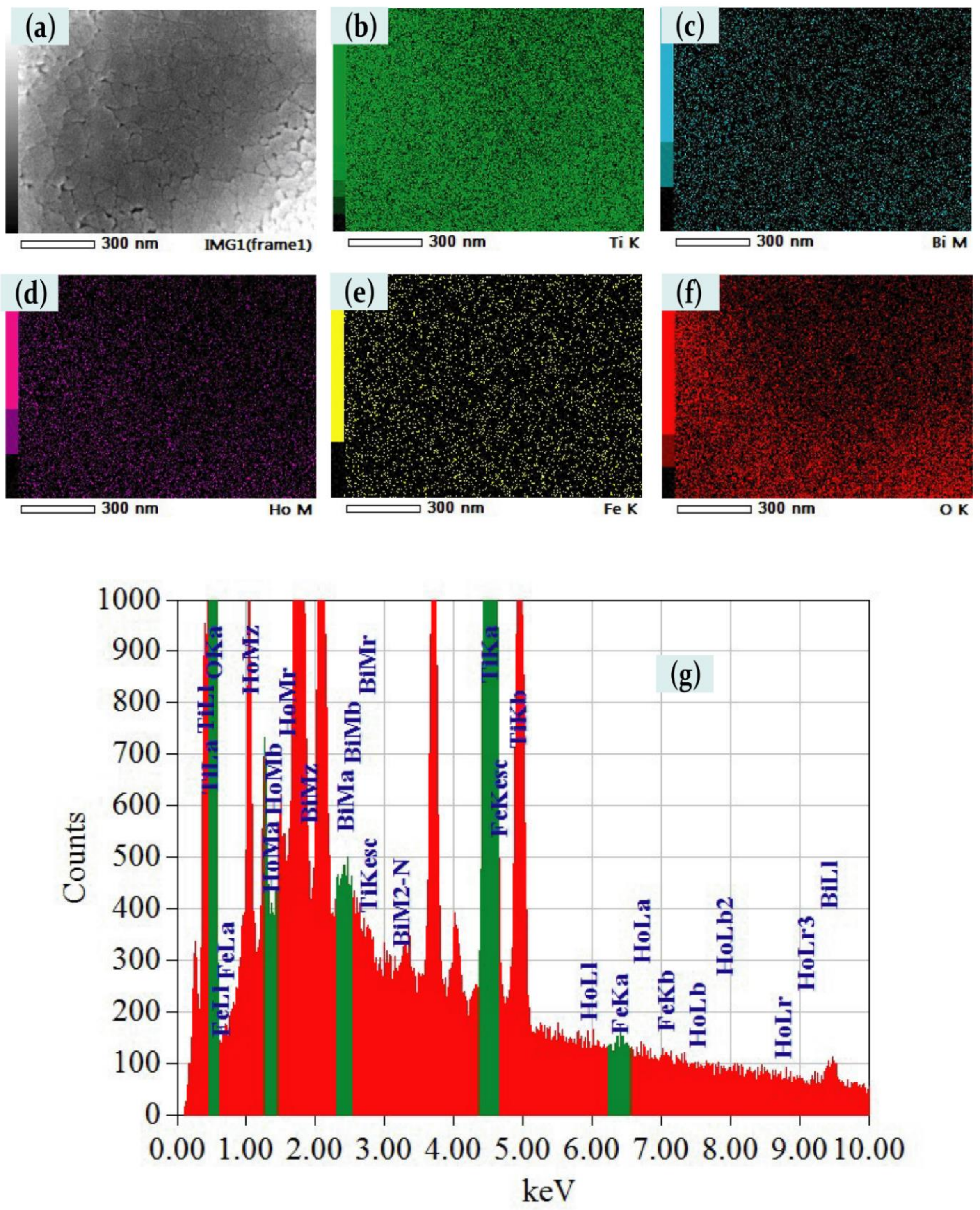

Figure S4. Elemental mapping of (b) Ti (green), (c) Bi (blue), (d) Ho (pink), (e) Fe (yellow), and (f) O (red) taken from the selected area of the FESEM image as shown in (a). (g) EDS pattern with the composition of the elements.

EDX studies

EDS analysis and elemental mapping were performed to gain insight on spatial distribution of elements, as shown in Fig. S4. Although the EDS spectrum and elemental mappings in Fig S4. b–g are exhibiting good homogeneous distribution of the elements (Ti (green), Bi (blue), Ho (pink), Fe (yellow), and O(red)), this homogeneous distribution could be attributed to large interaction volume produced during the EDS operation. An incident electron beam of 15 kV utilized during this operation was expected to penetrate a depth of 1 to 2 μm into the film in all directions.

Table S2: EDS analysis of TiO_2, $T^{95}B^{5}$ and $T^{80}B^{20}$ films

Film	Elements (Atom%)				
	Ti	O	Bi	Fe	Ho
TiO_2	18.95	81.05			
$T^{95}B^{5}$	43.33	50.69	3.04	2.28	0.66
$T^{80}B^{20}$	15.01	57.60	15.37	10.05	1.97

From EDS analysis it is also clear that $T^{95}B^{5}$ contains small atomic percentage of Bi, Fe and Ho. The percentage of these elements has increased in $T^{80}B^{20}$ film as the mol.% of BHFO has also increased.

Optical studies

The positions of conduction and valence band edge of semiconductors were calculated using electronegativity which is the geometric mean of the electronegativity of the constituent atoms:[8,9]

$$\chi_S = \sqrt[N]{\chi_1^a \chi_2^b \cdots \chi_{n-1}^p \cdot \chi_n^q} \qquad (1)$$

Where χ_n, q and N are the electronegativity of the constituent atom, the number of spices, and the total number of atoms in the compound respectively. Herein the electronegativity of an atom is the arithmetic mean of the atomic electron affinity (EA) and the first ionization potential (FIP):

$$\chi_n = \frac{FIP+EA}{2}$$

Table S3: EA, FIP, and calculated electronegativity for several atoms.

Elements	FIP (eV)	EA (eV)	Electronegativity, (χ_n) (eV)
Ti	6.826	0.078	3.452
O	13.618	1.4614	7.539
Bi	7.285	0.945	4.115
Ho	6.018	0.518	3.268
Fe	7.87	0.162	4.016

Using Supplementary Equation (1) and Table S3, the electronegativity of $Bi_{0.9}Ho_{0.1}FeO_3$,

$$\chi_{BHFO} = \sqrt[5]{(4.12)_{Bi}^{0.9}(3.268)_{Ho}^{0.1}(4.016)_{Fe}^{1}(7.539)_{O}^{3}} = 5.86$$

Similarly the electronegativity TiO_2 is found to be 5.81.

Magnetic studies

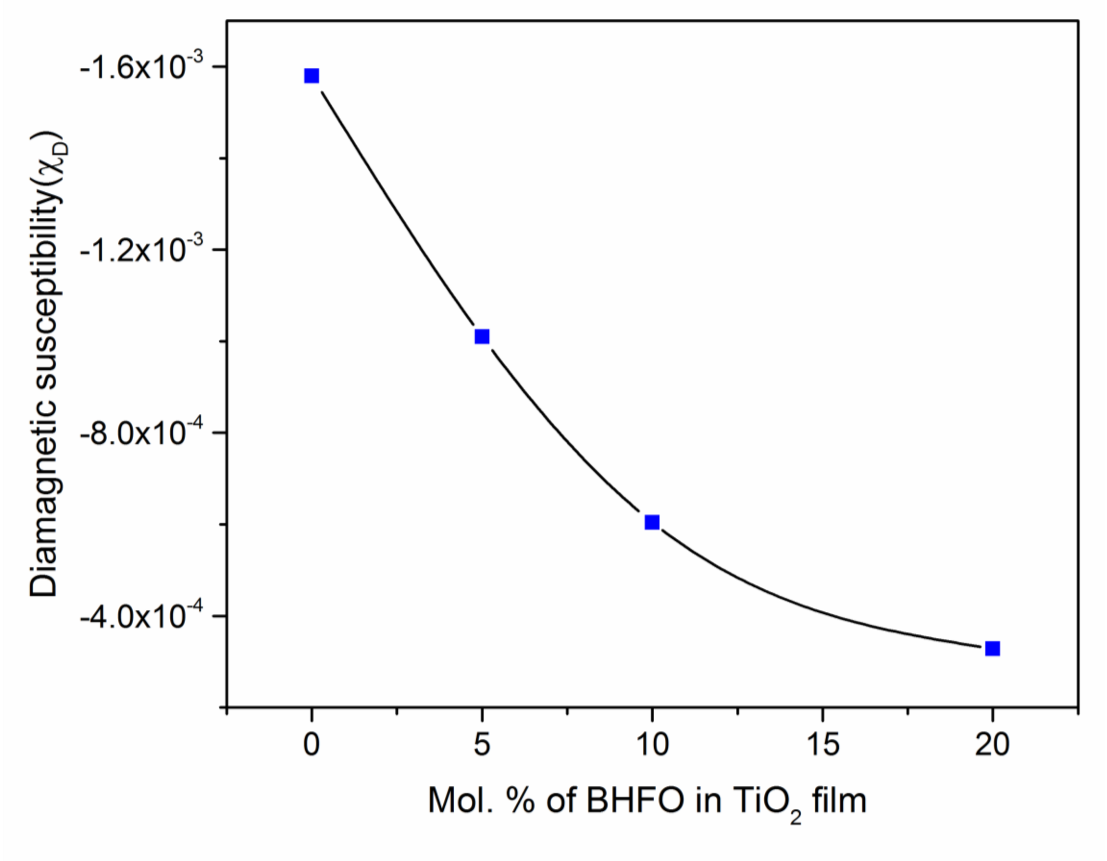

Figure S5. Diamagnetic susceptibility of films with different mol. % of BHFO nanoparticles

Reference

1 Hussain, S. *et al.* Size and lone pair effects on the multiferroic properties of Bi0. 75A0. 25FeO3−δ (A= Sr, Pb, and Ba) ceramics. *Journal of the American Ceramic Society* **96**, 3141-3148 (2013).

2 Jayakumar, O. *et al.* Theoretical and experimental evidence of enhanced ferromagnetism in Ba and Mn cosubstituted BiFeO 3. *Applied Physics Letters* **96**, 032903 (2010).

3 Khomchenko, V. *et al.* Intrinsic nature of the magnetization enhancement in heterovalently doped Bi1− xAxFeO3 (A= Ca, Sr, Pb, Ba) multiferroics. *Journal of Physics D: Applied Physics* **41**, 102003 (2008).

4 Qi, Y., Alima, B. & Shifeng, Z. Lattice distortion of holmium doped bismuth ferrite nanofilms. *Journal of Rare Earths* **32**, 884-889 (2014).

5 Hou, Z.-L. *et al.* Enhanced ferromagnetism and microwave absorption properties of BiFeO 3 nanocrystals with Ho substitution. *Materials Letters* **84**, 110-113 (2012).

6 Pradhan, S. & Roul, B. Effect of Gd doping on structural, electrical and magnetic properties of BiFeO3 electroceramic. *Journal of Physics and Chemistry of Solids* **72**, 1180-1187 (2011).

7 Rodriguez-Carvajal, J. in *satellite meeting on powder diffraction of the XV congress of the IUCr.* (Toulouse, France:[sn]).
8 Nethercot Jr, A. H. Prediction of Fermi energies and photoelectric thresholds based on electronegativity concepts. *Physical Review Letters* **33**, 1088 (1974).
9 Sanderson, R. Chemical Periodicity (Reinhold, New York, 1960). *Google Scholar*, 86 (1971).
